# Supplementary material for: AQuaRef: machine learning accelerated quantum refinement of protein structures
Source: Nat Commun. 2025 Oct 17;16:9224. doi: 10.1038/s41467-025-64313-1 (PMC12534518; doi:10.1038/s41467-025-64313-1)
Supplement: Supplementary file 1 — Supplementary Information [file 41467_2025_64313_MOESM1_ESM.pdf]

**Supplementary Table 1.** Refinement times for 41 cryo-EM models: Phenix refinement with Ramachandran plot, secondary structure, rotamer and standard restraints and AQuaRef.

| | | Time (minutes) | | |
|---|---|---|---|---|
| PDB code | Number of atoms | Phenix | AQuaRef | Ratio: Time AQuaRef / Time Phenix |
| 5xb1 | 2522 | 6 | 5 | 0.83 |
| 5yi5 | 2774 | 8 | 5 | 0.63 |
| 6ezj | 2851 | 5 | 5 | 1.00 |
| 6j6j | 1731 | 4 | 4 | 1.00 |
| 6wik | 13230 | 12 | 39 | 3.25 |
| 6xmx | 1976 | 5 | 4 | 0.80 |
| 6y9w | 6867 | 9 | 12 | 1.33 |
| 6y9x | 6867 | 8 | 12 | 1.50 |
| 7dnj | 5551 | 7 | 9 | 1.29 |
| 7k9i | 6441 | 7 | 11 | 1.57 |
| 7kzn | 1324 | 3 | 3 | 1.00 |
| 7lkh | 8236 | 9 | 14 | 1.56 |
| 7pcq | 4381 | 7 | 8 | 1.14 |
| 7un3 | 18922 | 15 | 38 | 2.53 |
| 7vvk | 17880 | 15 | 33 | 2.20 |
| 7vvn | 17338 | 14 | 34 | 2.43 |
| 7vxz | 14348 | 11 | 27 | 2.45 |
| 8aza | 9986 | 8 | 17 | 2.13 |
| 8ckz | 4513 | 7 | 7 | 1.00 |
| 8cl2 | 5440 | 7 | 9 | 1.29 |
| 8cl4 | 5440 | 7 | 9 | 1.29 |
| 8dat | 1226 | 3 | 3 | 1.00 |
| 8dl8 | 13419 | 18 | 25 | 1.39 |
| 8dq0 | 4753 | 7 | 8 | 1.14 |
| 8e6k | 9223 | 9 | 18 | 2.00 |
| 8esa | 6101 | 6 | 9 | 1.50 |
| 8fsj | 18915 | 13 | 39 | 3.00 |
| 8g94 | 18682 | 14 | 36 | 2.57 |
| 8idn | 6370 | 7 | 12 | 1.71 |
| 8jo4 | 11311 | 10 | 19 | 1.90 |
| 8qjx | 3038 | 6 | 6 | 1.00 |
| 8qjy | 3038 | 5 | 6 | 1.20 |
| 8qk3 | 3038 | 5 | 6 | 1.20 |
| 8r1f | 16668 | 13 | 30 | 2.31 |
| 8r1g | 16668 | 12 | 30 | 2.50 |
| 8sgj | 15271 | 11 | 27 | 2.45 |
| 8sgt | 14174 | 10 | 25 | 2.50 |
| 8ve0 | 1791 | 4 | 4 | 1.00 |
| 8vi2 | 4943 | 6 | 7 | 1.17 |

| | | | | |
|---|---|---|---|---|
| 8vi4 | 4942 | 6 | 7 | 1.17 |
| 8vi5 | 4942 | 6 | 9 | 1.50 |

**Supplementary Table 2.** List of 41 cryo-EM models and corresponding homologous high-resolution models with matching chains indicated.

| PDB code | Matching chain | Reference PDB code | Matching chain |
|---|---|---|---|
| 6j6j | A | 2f01 | A |
| 8dq0 | A | 2vw8 | A |
| 5xb1 | A | 4y08 | A |
| 8dat | K | 4xof | A |
| 7vvk | B | 5kdo | B |
| | G | 6crk | G |
| | P | 1et1 | A |
| 5yi5 | A | 4y08 | A |
| 7vvn | B | 5kdo | B |
| | G | 6crk | G |
| | P | 1et1 | A |
| 6xmx | A | 1r29 | A |
| 6y9x | A | 2pxr | C |
| | B | 6es8 | A |
| | N | 2xt1 | A |
| 6y9w | A | 2pxr | C |
| | B | 6es8 | A |
| | N | 2xt1 | A |
| 7kzn | K | 5e0l | A |
| 8ve0 | A | 4qxv | A |
| 8dl8 | C | 5kvg | L |
| 6ezj | A | 5ekw | A |
| 6wik | C | 5kvg | L |
| 7dnj | E | 4xof | A |
| | H | 4xof | A |
| 8aza | C | 4j44 | A |
| 7lkh | L | 2xkn | A |
| 7pcq | A | 1ird | A |
| | B | 1ird | B |
| 7k9i | L | 6frj | H |
| 7vxz | 3 | 1eaj | A |
| 7un3 | D | 4xof | A |
| 8esa | A | 3mre | A |
| | B | 1k5n | B |
| 8qjy | A | 3exv | A |

| | | | |
|---|---|---|---|
| 8ckz | A | 5w4o | A |
| | B | 2xt1 | A |
| 8cl2 | A | 5w4o | A |
| | B | 2xt1 | A |
| | C | 2xt1 | A |
| 8g94 | C | 5kdo | B |
| | D | 6crk | G |
| | E | 6crk | H |
| | G | 3hup | A |
| 8cl4 | A | 5w4o | A |
| | B | 2xt1 | A |
| | C | 2xt1 | A |
| 8idn | L | 6frj | H |
| 8e6k | A | 3b7e | A |
| 8fsj | B | 5i1k | L |
| | H | 6mee | A |
| | L | 6mee | B |
| 8r1g | B | 4xr8 | F |
| | C | 3d06 | A |
| 8qjx | A | 3exv | A |
| 8qk3 | A | 3exv | A |
| 8r1f | B | 4xr8 | F |
| | C | 3d06 | A |
| 8sgt | L | 6frj | H |
| 8sgj | L | 6frj | H |
| 8jo4 | B | 4b1y | B |
| 8vi5 | A | 3m71 | A |
| 8vi4 | A | 3m71 | A |
| 8vi2 | A | 3m71 | A |

**Supplementary Table 3.** List of 20 X-ray models and corresponding homologous high-resolution models with matching chains indicated.

| PDB code | Matching chain | Reference PDB code | Matching chain |
|---|---|---|---|
| 1fb5 | A | 1oth | A |
| 1jkt | A | 2w4j | A |
| | B | 2w4j | A |
| 1u87 | A | 6ji6 | A |
| 1wl3 | A | 1v4e | B |
| | B | 1v4e | B |
| 1xgo | A | 1xgs | B |
| 2fdq | A | 1hb6 | A |
| | B | 1hb6 | A |
| | C | 1hb6 | A |
| 2yhj | A | 3zix | F |
| | B | 3zix | F |
| 4xcr | A | 6flh | A |
| | B | 6flh | A |
| 4yei | A | 4yc5 | A |
| | B | 4yc5 | A |
| | C | 4yc5 | A |
| 1fp9 | A | 5jiw | A |
| 1m10 | A | 5bv8 | A |
| 1u9o | A | 5nio | A |
| | B | 5nio | A |
| 1w60 | A | 1u7b | A |
| | B | 1u7b | A |
| 1x24 | A | 5bx1 | A |
| | B | 5bx1 | A |
| 1yab | A | 1o6a | B |
| | B | 1o6a | B |
| 2a8z | A | 1h12 | A |
| 2etc | A | 2etb | A |
| | B | 2etb | A |
| 2h1g | A | 1st9 | B |
| | B | 1st9 | B |
| 2jcl | A | 1gx4 | B |
| | B | 1gx4 | B |
| 2pej | A | 2peq | B |

| | | | |
|---|---|---|---|
| | B | 2peq | B |
| | C | 2peq | B |
| | D | 2peq | B |
| | E | 2peq | B |
| | F | 2peq | B |

**Supplementary Table 4.** Refinement of 10 high-resolution X-ray structures at resolution 1 Å or better using Phenix and AQuaRef, showing $R_{work}$, $R_{free}$ and deviations of covalent bonds and angles from ideal library values.

| | | **Original** | | | | **Phenix refinement** | | | | **AQuaRef refinement** | | | |
|---|---|---|---|---|---|---|---|---|---|---|---|---|---|
| PDB | Resol. (Å) | Rwork | Rfree | Bonds (Å) | Angles (deg) | Rwork | Rfree | Bonds (Å) | Angles (deg) | Rwork | Rfree | Bonds (Å) | Angles (deg) |
| 2PND | 0.97 | 0.1526 | 0.1631 | 0.013 | 1.5 | 0.1192 | 0.1449 | 0.011 | 1.5 | 0.1156 | 0.1438 | 0.03 | 2.3 |
| 4JP6 | 1 | 0.1591 | 0.1857 | 0.022 | 1.9 | 0.1558 | 0.1825 | 0.009 | 1.3 | 0.1521 | 0.1832 | 0.027 | 2.2 |
| 4R5R | 0.96 | 0.1416 | 0.1583 | 0.026 | 2.1 | 0.144 | 0.1597 | 0.009 | 1.5 | 0.1415 | 0.16 | 0.026 | 2.3 |
| 4O8H | 0.85 | 0.1197 | 0.1314 | 0.015 | 1.7 | 0.1041 | 0.1167 | 0.009 | 1.5 | 0.1026 | 0.1166 | 0.018 | 1.9 |
| 6ZM8 | 0.78 | 0.1225 | 0.1339 | 0.014 | 1.8 | 0.126 | 0.1375 | 0.008 | 1.3 | 0.1243 | 0.139 | 0.019 | 1.9 |
| 5ZGL | 0.95 | 0.1058 | 0.124 | 0.013 | 1.5 | 0.0965 | 0.1207 | 0.012 | 1.6 | 0.0921 | 0.1156 | 0.019 | 2.9 |
| 6DKZ | 0.99 | 0.1729 | 0.1769 | 0.008 | 1.3 | 0.1712 | 0.1763 | 0.006 | 1.3 | 0.1688 | 0.1785 | 0.013 | 1.6 |
| 3NJW | 0.86 | 0.1113 | 0.1119 | 0.017 | 1.9 | 0.0959 | 0.1114 | 0.008 | 1.7 | 0.0933 | 0.1092 | 0.015 | 1.9 |
| 2FMA | 0.85 | 0.1378 | 0.1571 | 0.034 | 2.4 | 0.143 | 0.1573 | 0.012 | 1.4 | 0.1429 | 0.1592 | 0.031 | 1.8 |
| 1TT8 | 1 | 0.1209 | 0.1284 | 0.017 | 2.3 | 0.1245 | 0.1412 | 0.015 | 1.7 | 0.122 | 0.1413 | 0.029 | 2.6 |
